# One step ahead: timing and sexual networks in population mobility and HIV prevention and care

**DOI:** 10.1002/jia2.25140

**Published:** 2018-07-19

**Authors:** Susan Cassels, Carol S Camlin, Janet Seeley

**Affiliations:** ^1^ Department of Geography University of California Santa Barbara CA USA; ^2^ Department of Obstetrics, Gynecology and Reproductive Sciences University of California San Francisco CA USA; ^3^ Department of Medicine University of California San Francisco CA USA; ^4^ Department of Global Health and Development London School of Hygiene and Tropical Medicine London UK

**Keywords:** time, networks, universal test and treat, bridging, sexual risk behaviour, HIV care cascade

## 1 Introduction

Population mobility comes in heterogeneous forms and is triggered by many drivers. The diverse contexts of mobility can significantly influence the effectiveness of HIV prevention and care, as the contributions to this volume highlight. Nevertheless, some fundamental concepts are common across various forms of mobility. Two such concepts are time and sexual networks: mobility occurs in a space‐time continuum, and migrants are situated within social and sexual networks. In this viewpoint, we argue that a closer examination of how (1) time scales of mobility and (2) sexual network characteristics of migrants present challenges to effective HIV prevention can help to optimize interventions.

## 2 Time

We must consider time as well as space when conceptualizing and examining how migration might affect HIV prevention and care, including Universal Test and Treat (UTT) interventions. Forms of population mobility, especially in sub‐Saharan Africa, are complex and often characterized by multiple rounds of travel, seasonal migration 1 or movement events in time 2, 3, 4, 5. Circular migration, where migrants leave home to work (or for other reasons) but frequently return home before leaving again, is also common, especially in South Africa 6, 7. Sexual risk behaviour of circular migrants vary over time as well: before migrating, while away and after returning home 8, 9.

Basic concepts of epidemiology will predict an association between migration and the effect of an intervention if we assume a dose‐response relationship: the more the exposure to an effective HIV intervention, the larger the response. Thus, the timing of migration and chances of exposure to interventions matter for effective outcomes. For example, during a community trial of UTT, a circular migrant will not be exposed to the same level of messaging and linkage to care efforts compared with non‐migrants in their place of origin 10. Additionally, treatment and prevention services may not reach migrants arriving in a new destination. Campaigns to increase HIV testing may miss new arrivals; they may not know where to get tested in an unfamiliar place, or may face political and structural barriers to care, such as those related to legal documentation status 11.

A recent longitudinal study in Uganda 12 found that HIV incidence decreased for permanent residents over time and scale‐up of combination prevention efforts, but the same decline was not observed for migrants who had recently arrived. Similar evidence emerged in fishing villages in Uganda, where individuals who had been in the community for less than five years showed higher rates of seroconversion than longer term residents 13.

Migrants often exhibit riskier sexual behaviour while away from home compared with non‐migrants 14, 15, 16, possibly due to an enabling environment. Moreover, behaviours of migrants vary with respect to *timing* of migration events as well, and seemingly converge to levels of risk behaviour of non‐migrants over time 12. Therefore, the timing of engagement into HIV care for migrants may be doubly important. Because of the non‐linear nature of HIV transmission dynamics and potential for engagement in risky behaviour, a migrant disengaged from HIV care could contribute to a disproportionate amount of ongoing HIV transmission over space and time 17.

Time is also important when considering the particular stage of a person's life in which migration takes place. Following a life‐course approach 18, a younger woman may be confronted by challenges to prevention and care that are different from those of an older woman, or indeed a man of any age. A migrant's life is situated within social relationships, and the social timing of mobility, such as moving when a single woman, a parent or a widow, impacts behaviour. For example, a single woman may face particular challenges because being seen as alone and available; travelling as a mother without her children, she may be anxious about their care and safety. If she travels with children, she will need to find safe accommodation and time to care for them. Mobility is also situated in historical time: when someone moves, it exposes that person to constraints and opportunities that may differ from those of someone moving to the same place a decade earlier or later (this is termed, in demography, a “cohort effect”). Finally, there are variations in the extent people can influence the course of their life through the choices they make about mobility, sexual behaviour, prevention and care. If a young woman is struggling to find work in a place she has moved to, she may turn to transactional sex to gain access to food or shelter; this may make her vulnerable to sexually transmitted infections, violence and abuse. Human agency can be influenced by many factors: gender, age, socio‐economic status, where one comes from and where one moves 19, 20.

## 3 Sexual networks

Sexual transmission of HIV occurs within structured sexual networks 21, 22, 23 and the characteristics of sexual networks can influence the effectiveness of UTT. For migrants, the period after migration is often associated with instability and detachment from family, friends and previous community, with fewer constraints from social norms governing sexual behaviours 24, 25. The structure and context of migrant sexual networks are critical for understanding risks of HIV transmission 26 and the effectiveness of prevention interventions: the location (place) and timing of sexual ties can interrupt or dilute the effectiveness of interventions such as UTT. Migrant networks can bridge otherwise separate places and contribute to ongoing HIV transmission by engaging in sex acts in different places or maintaining relationships with sexual partners who live in different places 27. The reverse is also possible, but less examined: migration can bridge places with different UTT coverage and interrupt the effectiveness of interventions. For example, large flows of migrants arriving in a new destination could reduce the proportion of a population on antiretroviral therapy below a critical threshold so that HIV could continue to circulate. Therefore, public health surveillance needs to account for both permanent and temporary migration flows.

The timing and sequence of migrant's sexual partnerships is also an important factor in anticipating and mitigating the effects of mobility on HIV prevention and care. Typically, individuals choose sexual partnerships with people who are similar to them (e.g. age, race/ethnicity) 28. Sexual partners of mobile individuals may also be migrants or people living outside their home community (assortative mixing by migration status) 14, 27. Therefore, a migrant's partners may lack exposure to the same level of HIV treatment and prevention, and thus have higher rates of HIV infection. The spatial and temporal structure of sexual networks may result in a lower impact (e.g. population‐level incidence) given the same amount of effort/intervention.

## 4 One step ahead

As evidenced by the articles in this supplement, population mobility is complex. Nevertheless, there exist some fundamental concepts that we can rely on to understand when and why mobility presents challenges for HIV prevention and care, and how to improve interventions. Since timing of mobility can influence the effectiveness of HIV prevention efforts, the rollout of interventions needs to account for time and potential missing populations. For example, seasonal patterns of mobility can be used to strategize and optimize interventions. For example, in Niger, seasonal migration patterns were estimated with satellite imagery in order to predict the most effective time for a measles vaccination campaign 29. Second, spatial and temporal characteristics of migrant sexual networks may reduce intervention effectiveness, but a network perspective can be leveraged to improve or broaden interventions as well. For instance, mobile individuals can distribute HIV self‐tests or deliver antiretroviral drugs to partners, possibly obtaining a broader coverage of testing or treatment as prevention than otherwise possible with individual interventions. Knowledge of the mobility of specific populations in specific settings can be used to inform, fine‐tune, and thus amplify the potential effectiveness of differentiated care models as well as HIV prevention interventions. Such interventions are urgently needed to enable migrants to maintain their health and that of their sexual partners 30.
